# Association between fast eating speed and metabolic dysfunction-associated steatotic liver disease: a multicenter cross-sectional study and meta-analysis

**DOI:** 10.1038/s41387-024-00326-x

**Published:** 2024-08-14

**Authors:** Miao Zhang, Xiaoyang Sun, Xiaopeng Zhu, Lili Zheng, Yufang Bi, Qiang Li, Lirong Sun, Fusheng Di, Yushan Xu, Dalong Zhu, Yanyan Gao, Yuqian Bao, Yao Wang, Lanjie He, Chenmin Fan, Xin Gao, Jian Gao, Mingfeng Xia, Hua Bian

**Affiliations:** 1 Department of Endocrinology and Metabolism, Zhongshan Hospital, Fudan University, Shanghai, China; 2 Fudan Institute for Metabolic Disease, Fudan University, Shanghai, China; 3 Department of Endocrinology and Metabolism, The First Affiliated Hospital of Zhengzhou University, Zhengzhou, China; 4 Shanghai National Clinical Research Center for Endocrine and Metabolic Diseases, Key Laboratory for Endocrine Tumors of Ministry of Shanghai, Shanghai Institute for Endocrine and Metabolic Diseases, Ruijin Hospital, Shanghai Jiaotong University School of Medicine, Shanghai, China; 5 Department of Endocrinology and Metabolism, The Second Affiliated Hospital of Harbin Medical University, Harbin, China; 6 Department of Endocrinology and Metabolism, Shenzhen University General Hospital, Shenzhen, China; 7 Key Laboratory of Hormones and Development (Ministry of Health), Tianjin Key Laboratory of Metabolic Diseases, Tianjin Metabolic Diseases Hospital and Tianjin Institute of Endocrinology, Tianjin Medical University, Tianjin, China; 8 Department of Endocrinology and Metabolism, The Third Central Hospital of Tianjin, Tianjin, China; 9 Department of Endocrinology and Metabolism, The First Affiliated Hospital of Kunming Medical University, Kunming, China; 10 Department of Endocrinology and Metabolism, Drum Tower Hospital Affiliated to Nanjing University Medical School, Nanjing, Jiangsu China; 11 Department of Endocrinology and Metabolism, Affiliated Hospital of Medical College, Qingdao University, Qingdao, China; 12 Shanghai Diabetes Institute, Shanghai Key Laboratory of Diabetes Mellitus, Shanghai Clinical Center for Diabetes, Department of Endocrinology and Metabolism, Shanghai Jiaotong University Affiliated Sixth People’s Hospital, Shanghai, China; 13 Department of Endocrinology and Metabolism, Zhongda Hospital Affiliated to Southeast University Medical School, Nanjing, China; 14 Endocrine Testing Center, General Hospital of Ningxia Medical University, Yinchuan, China; 15 Department of Endocrinology, Qilu Hospital of Shandong University, Qingdao, China; 16 Department of Clinical Nutrition, Zhongshan Hospital, Center of Clinical Epidemiology, EBM of Fudan University, Fudan University, Shanghai, China; 17 Center of Clinical Epidemiology and Evidence-Based Medicine, Fudan University, Shanghai, China

**Keywords:** Risk factors, Metabolic syndrome

## Abstract

**Background:**

With the fast pace of modern life, people have less time for meals, but few studies have examined the association between the habit of fast eating and metabolic diseases.

**Objective:**

Combining the results of the current study and the prior ones, we aimed to investigate the possible relationship between fast eating and the risk of metabolic dysfunction-associated steatotic liver disease (MASLD).

**Methods:**

This is a sub-analysis of a multicenter cross-sectional study of 1965 participants investigated the association between fast eating and MASLD in Chinese. Fast eating was defined as meal time less than five minutes and participants were divided into three categories based on their self-reported frequency of fast eating: ≤1 time/month, ≤1 time/week and ≥2 times/week. We further conducted a literature search for available studies published before November, 2023 as well as a meta-analysis to investigate the association between fast eating and MASLD.

**Results:**

The proportion of MASLD was 59.3%, 50.5%, and 46.2% in participants with fast eating ≥2 times/week, ≤1 time/week and ≤1 time/month, respectively (P for trend <0.001). The frequency of fast eating was independently associated with risk of MASLD after multiple adjustment for sex, age, demographics, smoking and drinking status, BMI and clinical metabolic parameters (OR, 1.29; 95%CI, 1.09–1.53). Participants who ate fast frequently (≥2 times/week) had 81% higher risk of MASLD (*P* = 0.011). A meta-analysis of five eligible studies confirmed that frequent fast eating was associated with increased risk of MASLD (pooled OR, 1.22; 95%CI, 1.07–1.39).

**Conclusions:**

Frequent fast eating was associated with an increased risk of MASLD.

## Introduction

Nonalcoholic fatty liver disease (NAFLD) is a common liver disease characterized by excessive hepatic fat accumulation without excessive alcohol consumption, which can potentially lead to liver cirrhosis and hepatocarcinoma [1]. In 2023, a broad consensus proposed a nomenclature change from NAFLD to metabolic dysfunction-associated steatotic liver disease (MASLD) [2] to stress the pathophysiology of the disease, while studies suggested that NAFLD and MASLD definitions are identical in terms of criteria, clinical profiles and natural histories [3, 4]. Thus, previous research results on NAFLD provide main evidence when investigating MASLD. MASLD is closely associated with obesity, metabolic syndrome, hypertension and diabetes [1, 5]. Lifestyle intervention remains the only recommended treatment for MASLD, and weight loss of 7–10% can effectively reduce liver steatosis and inflammation [6].

Many people eat fast under the fast-paced modern life. However, several previous studies indicated that eating at fast speed, herein reported “fast eating”, was an unhealthy habit that contributed to multiple metabolic disorders [7–9], especially overweight and obesity [10, 11]. Individuals who ate fast were more likely to suffer from glycemic excursion [12] and develop newly-onset diabetes [13]. Furthermore, fast eating was also associated with elevated alanine aminotransferase (ALT) according to previous studies [14]. However, the association between the habit of eating fast and risk of MASLD is still under controversy. Several studies reported that patients with MASLD ate faster than those without MASLD [15–17]. Lee et al. found the fast eaters had higher risk of MASLD among Korean population [18]. A correlation between fast eating and MASLD was also reported in male diabetic patients [19]. However, studies have also reported none independent association between fast eating and MASLD [20, 21], including a previous study among Chinese adults that found no significant correlation between fast eating and risk of incident MASLD after adjustment for body mass index (BMI) and waist circumference (WC) [22].

In this multicenter cross-sectional study, we examined the association between the frequency of fast eating and the risk of MASLD in 1965 adults enrolled from 10 clinics of obesity, diabetes and metabolic disease located in six provinces/municipalities in China. Moreover, a meta-analysis was conducted by combining the available data from previous studies and our current study to further investigate the association between fast eating and the risk of MASLD.

## Methods

### Cross-sectional study

This study recruited 2704 participants from 10 clinics of obesity, diabetes, and metabolic disease at six provinces/municipalities in China from January 2011 to December 2011, as detailed in our previous study [23]. Each participant was given a unique clinical ID on an online registration system to ensure continuous enrollment. After excluding the participants with lack of necessary data, excess alcohol consumption (>20 g/day for men, >10 g/day for women), presence of viral hepatitis, other known liver diseases or endocrine disorders, a total of 1965 eligible subjects were eventually included in the analysis (Fig. S1).

Demographic information, past history of cigarette smoking and alcohol drinking, medical history and current medications were collected through face-to-face interviews using a standardized questionnaire. Based on the Qinling–Huaihe Line, a reference line distinguishing South and North China, participants were divided into Southern (Shanghai, Jiangsu, and Henan) and Northern (Heilongjiang, Tianjin, and Shandong) groups. Data on height, weight, blood pressure (BP) and WC were acquired through a physical examination. BMI was then calculated as the weight (kg) divided by height^2^ (m^2^).

Blood samples were collected after an overnight fast of at least 10 h for biochemical examination. Serum triglycerides (TG), total cholesterol (TC), ALT, aspartate aminotransferase (AST), gamma glutamyl transferase (GGT), low-density lipoprotein cholesterol (LDL-c), high-density lipoprotein cholesterol (HDL-c) and uric acid (UA) were measured using a 7600 Automatic Biochemical Analyzer (Hitachi Ltd, Tokyo, Japan). Hemoglobin A1c (HbA1c) was measured by high-performance liquid chromatography (HPLC) (BIO‐RAD II TURBO). A 75 g oral glucose tolerance test was performed to measure fasting blood glucose (FBG) and 2-hour postload blood glucose (2hPBG) of each subject, according to which the subjects were then divided into three groups of glucose metabolism status: type 2 diabetes mellitus (T2DM), impaired glucose regulation (IGR) and normal glucose tolerance (NGT).

Fatty liver was defined under liver ultrasonography according to the same criteria: increased liver echogenicity in contrast to renal cortex and portal vein blurring [24]. All ultrasound tests were performed by experienced sonographers with 4 MHz probe on fixed machines, which were all calibrated by a 3D abdominal phantom (Model 057A; CIRS - Computerized Imaging Reference Systems Inc., Virginia, USA) for consistency.

An eating habit frequency questionnaire was conducted through face-to-face interviews, including items of skipping breakfast, eating before bed, snacking and fast eating. Fast eating was defined as the time taken to eat less than five minutes. Based on self-assessment, the participants were required to choose from three frequency categories including “≤1 time/month”, “≤1 time/week” and “≥2 times/week”.

### Meta-analysis

We performed literature searches in PubMed and Web of Science for studies published before November 2023. The primary search was based on the following terms: (“eating speed” OR “speed of eating” OR “fast eating” OR “eating fast” OR “quick eating” OR “rapid eating”) AND (“fatty liver” OR NAFLD OR nonalcoholic fatty liver disease OR liver steatosis OR steatohepatitis OR MASLD OR metabolic dysfunction-associated steatotic liver disease OR MAFLD OR metabolic associated fatty liver disease).

We included cross-sectional, cohort or case–control studies presenting odds ratio (OR) or relative risk (RR) to determine the association between the eating speed and risk of MASLD. After review of titles, abstracts and full texts, seven articles were selected from the initial pool for detailed evaluation and four eligible ones were included in the meta-analysis. The selection process was outlined in Fig. S2 employing the Preferred Reporting Items for Systematic Reviews and Meta-Analyses (PRISMA) diagram. The quality of the reviewed studies was assessed using the Newcastle-Ottawa Scale (NOS) and Agency for Healthcare Research and Quality (AHRQ) scale by two authors (M.Z. and X.S.) independently. All disagreements were resolved by consensus of the two authors.

### Statistical analysis

The continuous variables were presented as mean ± SD, except for the skewed ones that were presented as the median with the interquartile range (25–75%). The categorical variables were presented as counts and relative frequencies given in parentheses. Intergroup comparison was conducted using the one-way ANOVA or Wilcoxon tests where appropriate. Chi-squared tests were used for the categorical variables. The association between fast eating and MASLD, both overall and stratified, were assessed by multivariable logistic regression models. In Model 1, age, sex, smoking and drinking status, BMI and geographical region were adjusted. In Model 2, confounding variables including WC, FBG, HbA1c, TG, TC, GGT, LDL-c, HDL-c and UA were further adjusted in addition to the variables in Model 1. Potential interactions between fast eating and sex, geographical region, smoking status, glucose status, and BMI were assessed by log likelihood ratio test in stratified analyses.

For all studies included in the meta-analysis, participants were divided into two or more categories according to their eating speeds, and the slow eating groups were used as the reference group. We retrieved the adjusted ORs and 95%CIs of all non-slow-eating groups from the reviewed articles, which was then combined with our results to calculate the pooled OR and 95% CIs in the meta-analysis. The results were presented in forest plots. The heterogeneity among studies was calculated using the I^2^ and τ^2^ statistic and a random effect model was used. A sensitivity analysis was conducted by deleting one study at a time and recalculating combined estimates.

Statistical analyses were all performed with R version 4.2.3 statistical software (packages rms, epiDisplay, dplyr, ggplot2, PMCMRplus, metafor, forestploter). All results were 2-sided and *P* < 0.05 was regarded statistically significant.

## Results

### Cross-sectional study

Of the 1965 participants (977 men and 988 women), 45.6% were from the Northern part of China and 48.8% were diagnosed as MASLD. They had an average age of 53.6 years and a BMI of 24.80 ± 3.61 kg/m^2^ (Table S1). As shown in Table 1, participants who habitually ate fast tended to be smokers (*P* = 0.006) and alcohol-drinkers (*P* <0.001), and were more likely from the Northern part of China (*P* < 0.001). The proportion of the MASLD patients in three groups (fast eating at the frequency of ≤1 time/month, ≤1 time/week, ≥2 times/week) was 46.2%, 50.5%, and 59.3%, respectively (P <0.001). There is a significant difference in WC (*P* = 0.008), SBP (*P* = 0.036), ALT (*P* < 0.001), AST (*P* = 0.001), and GGT (*P* < 0.001) among the three groups. The participants with fast eating ≥ 2 times/week showed significantly higher ALT, AST,and GGT than those with fast eating ≤1 time/month, and higher BMI and SBP than the group with fast eating ≤1 time/week.

**Table 1:** Participants characteristics by fast eating frequency.

Characteristics^a^	Categories of fast eating frequency			*P*-value^b^
	≤1 time/mo (*n* = 1362)	≤1 time/wk (*n* = 313)	≥2 times/wk (*n* = 290)	
MASLD, *n* (%)	629 (46.2)	158 (50.5)	172 (59.3)	<0.001
Women, *n* (%)	699 (51.3)	144 (46.0)	145 (50.0)	0.236
Age, yr	53.04 ± 13.75	51.23 ± 12.83^*^	53.26 ± 13.95	0.087
Smoker, *n* (%)	353 (25.9)	87 (27.8)	102 (35.2)	0.006
Alcohol-drinker, *n* (%)	273 (20.0)	70 (22.4)	103 (35.5)	<0.001
Glucose metabolism, *n* (%)				0.087
NGT	342 (25.1)	78 (24.9)	74 (25.5)	
IGR	178 (13.1)	50 (16.0)	55 (19.0)	
T2DM	842 (61.8)	185 (59.1)	161 (55.5)	
North China, *n* (%)	490 (36.0)	197 (62.9)	209 (72.1)	<0.001
BMI (kg/m²)	24.83 ± 3.64	24.41 ± 3.43	25.06 ± 3.68^†^	0.085
SBP, mmHg	130.64 ± 19.90	127.67 ± 18.52^*^	131.21 ± 19.43^†^	0.036
DBP, mmHg	80.37 ± 11.32	80.88 ± 10.73	80.04 ± 11.62	0.647
WC, cm	88.43 ± 10.91	86.51 ± 9.81^*^	87.14 ± 10.88	0.008
HbA1c, %	6.80 (5.80–9.00)	7.15 (5.90–9.10)	7.20 (5.90–8.80)	0.169
FBG, mmol/L	6.15 (5.30–7.90)	5.98 (5.12–7.60)	6.20 (5.36–8.21)	0.156
2hPBG, mmol/L	11.80 (7.19–16.80)	10.74 (6.88–15.92)	10.40 (6.70–16.05)	0.105
TC, mmol/L	4.94 ± 1.26	4.84 ± 1.45	4.86 ± 1.20	0.346
TG, mmol/L	2.01 ± 2.01	1.98 ± 2.32	2.23 ± 2.91	0.263
HDL-c, mmol/L	1.24 ± 0.40	1.21 ± 0.41	1.27 ± 0.33	0.242
LDL-c, mmol/L	2.86 ± 0.96	2.76 ± 0.96	2.89 ± 0.85	0.172
ALT, U/L	18.00 (12.00–26.09)	20.00 (14.00–31.00)^*^	19.00 (14.00–28.00)^*^	<0.001
AST, U/L	20.00 (15.00–26.00)	21.00 (15.50–29.00)^*^	21.00 (17.00–27.00)^*^	0.001
GGT, U/L	25.00 (16.00–45.00)	31.00 (18.06–56.25)^*^	30.00 (17.00–54.00)^*^	<0.001
UA, μmol/L	295.10 ± 97.26	297.34 ± 105.35	304.41 ± 91.61	0.340

*MASLD* metabolic dysfunction-associated steatotic liver disease, *NGT* normal glucose tolerance, *IGR* impaired glucose regulation, *T2DM* type 2 diabetes mellitus, *BMI* body mass index, *SBP* systolic blood pressure, *DBP* diastolic blood pressure, *WC* waist circumference, *HbA1c* glycated hemoglobin, *FBG* fasting blood glucose, *2hPBG* 2-h postload blood glucose in oral glucose tolerance test, *TC* total cholesterol, *TG* triglyceride, *HDL-c* high density lipoprotein cholesterol, *LDL-c* low density lipoprotein cholesterol, *ALT* alanine aminotransferase, *AST* aspartate aminotransferase, *GGT* gamma-glutamyl transferase, *UA* uric acid.

^a^means ± SD or median (25th–75th percentile) or *n* (%), as appropriate.

^b^Analysis of variance, Kruskal–Wallis test or Chi-squared test.

^*^P <0.05 in comparison with ≤1 time/mo group, using LSD test or Tukey-Kramer-Nemenyi all-pairs test.

^†^*P* <0.05 in comparison with ≤1 time/wk group, using LSD test or Tukey-Kramer-Nemenyi all-pairs test.

The association between fast eating and MASLD risk was summarized in Table 2. The group with fast eating ≤1 time/month was used as a reference in all multivariable models. ORs (95% CIs) for MASLD were 1.20 (0.91–1.60) and 1.45 (1.08–1.96) in those who ate fast ≤1 time/week and ≥2 times/week, respectively, after adjustment for sex, age, cigarette-smoking, alcohol-drinking, geographical region and BMI. The association between fast eating ≥2 times/week and risk of MASLD remained significant after additional adjustment for all confounding metabolic parameters (WC, HBA1C, FBG, TC, TG, HDL-C, and LDL-C) and liver enzymes (OR, 1.81; 95%CI, 1.26–2.59).

**Table 2:** Overall ORs (95% CIs) for MASLD according to frequency categories of fast eating.

Logistic regression models	Categories of fast eating frequency			Fast eating frequency^c^
	≤1 time/mo (*n* = 1362)	≤1 time/wk (*n* = 313)	≥2 times/wk (*n* = 290)	
Unadjusted ORs (95%CIs)	Reference	1.19 (0.93–1.52)	1.70 (1.31–2.20)	1.28 (1.14–1.45)
*P* value		0.17	< 0.001	<0.001
Model 1^a^ ORs (95%CIs)	Reference	1.20 (0.91–1.60)	1.45 (1.08–1.96)	1.20 (1.04–1.39)
*P* value		0.199	0.015	0.011
Model 2^b^ ORs (95%CIs)	Reference	1.08 (0.78–1.49)	1.81 (1.26–2.59)	1.29 (1.09–1.53)
*P* value		0.661	0.011	0.003

*BMI* body mass index, *WC* waist circumference, *HbA1c* glycated hemoglobin, *FBG* fasting blood glucose, *2hPBG* 2-h postload blood glucose in oral glucose tolerance test, *TC* total cholesterol, *TG* triglyceride, *HDL-c* high-density lipoprotein cholesterol, *LDL-c* low-density lipoprotein cholesterol, *ALT* alanine aminotransferase, *AST* aspartate aminotransferase, *GGT* gamma-glutamyl transferase, *UA* uric acid.

^a^Model 1: Adjusted for age, sex, smoking status, alcohol-drinking status, BMI and region.

^b^Model 2: Adjusted for age, sex, smoking status and drinking status, region, BMI, WC, HbA1c, FBG, TC, TG, HDL-c, LDL-c. ALT, AST, GGT, UA.

^c^Fast eating frequency as a continuous variable in the models.

**Fig. 1:**
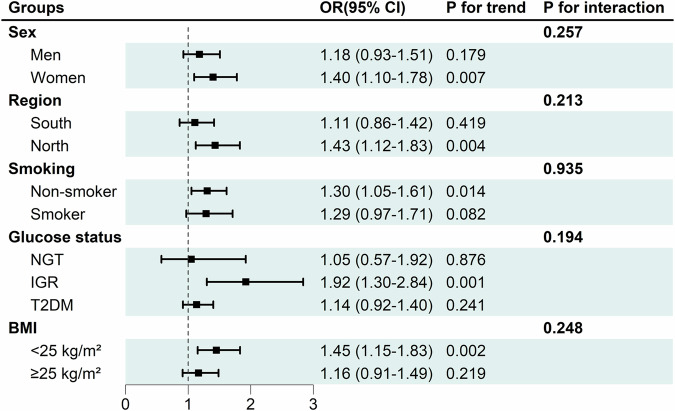
Stratified ORs (95% CIs) for MASLD according to frequency of fast eating. Fast eating frequency as a continuous variable in the model. Adjusted for age, sex, smoking status and drinking status, region, BMI, WC, HB1AC, FBG, TC, TG, HDL-C, LDL-C. ALT, AST, GGT, UA. NGT, normal glucose tolerance; IGR, impaired glucose regulation; T2DM, type 2 diabetes mellitus; BMI, body mass index; WC, waist circumference; HbA1c, glycated hemoglobin; FBG, fasting blood glucose; 2hPBG, 2-h postload blood glucose in oral glucose tolerance test; TC, total cholesterol; TG, triglyceride; HDL-c, high-density lipoprotein cholesterol; LDL-c, low-density lipoprotein cholesterol; ALT, alanine aminotransferase; AST, aspartate aminotransferase; GGT, gamma-glutamyl transferase; UA, uric acid.

Subgroup analyses were performed on the participants divided by sex, geographical region, cigarette smoking status, glucose metabolism status and BMI levels (Fig. 1 and Table S2), There was an independent association between the frequency of fast eating and risk of MASLD in women (*P* for trend = 0.007), non-smokers (*P* for trend = 0.014), northern dwellers (*P* for trend = 0.004), the participants with IGR (*P* for trend = 0.001) and BMI <25 kg/m² (*P* for trend = 0.002) after full adjustment for multiple confounders. No interaction was found between fast eating and major risk factors including sex, geographical region, smoking status, glucose status and BMI.

### Meta-analysis

We further conducted a meta-analysis to investigate the association between fast eating and the risk of MASLD. As shown in Fig. S1, A total of 25 articles were yielded from two databases. After removing the duplicated literature, 15 articles were under evaluation, of which six were considered irrelevant to this study and two were not written in English. In the seven reports qualified for review, we further removed two articles without sufficient adjustments in the regression models and one article conducted in diabetes patients, remaining four articles included for meta-analysis.

The characteristics of qualified studies were summarized in Table S3. Of the seven studies, one was retrospective cohort study [20] while six were cross-sectional studies [17–19, 21, 22, 25]. One of the studies were conducted in T2DM patients [19], while others recruited subjects in general population. All the reviewed articles were from Asian countries: four in Japan [19–21, 25], one in China [22], one in South Korea [18], and one in Iran [17]. Four studies used two categories to describe the eating speed variation [17, 20, 21, 25], two studies provided four categories [18, 22] and one study used three categories [19]. Two studies calculated Fatty Live Index (FLI) score [20] and Liver/spleen (L/S) attenuation ratio [21] as indicators for MASLD, while in other reports, MASLD was diagnosed under sonographic examination. Most studies adjusted covariates for sex, age, BMI, demographics and biochemical variables except for two studies; one study that applied univariate logistic regression model [17] and one study that applied multivariate regression model that adjusted only for age [25].

**Fig. 2:**
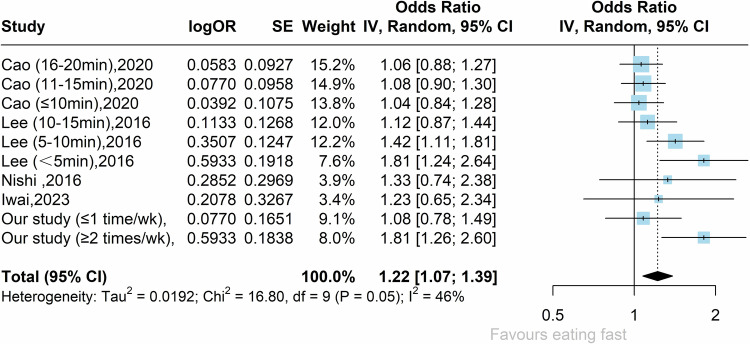
Association of eating speed and MASLD risk across studies. Forest plot for fast eating as a risk factor for MASLD using random-effects meta-analysis. The square size represents the weights of included estimates, the diamond indicates the overall risk estimate and black line segments indicate the 95% CI of each study. Heterogeneity between studies is shown by *I*^2^.

The meta-analysis of four qualified studies together with our results reported a pooled OR of 1.22 (95% CI: 1.07–1.39), as shown in the forest plot presented in Fig. 2. A substantial heterogeneity between studies was reported (*I*^2^ = 46%, *τ*^2^ = 0.0192, *P* = 0.05).

The overall quality of the reviewed studies was evaluated as moderate to high. As shown in Table S4, the NOS scores of the included studies ranged from 6 to 8. The six cross sectional studies scored 6 to 9 points in AHRQ scale (Table S5) and were of medium or high quality. Additionally, the result of leave-one-out sensitivity analysis was shown in forest plot in Fig. S3. The overall results remained significant every time one of the studies was removed.

## Discussion

Our findings in this multicenter cross-sectional study suggested that frequent fast eating was independently associated with a higher risk of developing MASLD. Further meta-analysis of four qualified studies together with our current study confirmed the association between fast eating and MASLD.

Although the mechanism behind the link between fast eating and MASLD has not been fully elucidated, it is conceivable that fast eating may cause excessive energy intake, resulting in weight gain and MASLD [26]. Eating speed is an important factor influencing energy intake [25, 26]. Compared to faster eaters, eating at a slow pace leads to a higher concentration of anorexigenic gut peptides and a more pronounced earlier satiety [27]. Moreover, fast eating group reported less fullness after the meal and less ghrelin suppression [28], probably leading to excessive food consumption [29]. Several previous studies have indicated that eating speed may influence body weight and waist circumference [10, 30–32] in adults, adolescents [33] and children [34, 35]. Kolay et al. [10] and Yuan et al. [36] have confirmed the effect of fast eating on overweight and metabolic syndrome in their meta-analyses, respectively, and the latter also examined the correlation between fast eating and various components of metabolic syndrome. Therefore, Cao et al. [22] claimed that the relationship between fast eating and MASLD was mediated by the changes in body weight, and the increased risk of MASLD found in the frequent fast eating group was no longer significant after adequate adjustment for confounding variables as reported previously [17, 19, 21, 22, 25].

Nevertheless, several studies [18] reported the association between fast eating and MASLD remained significant even after adjustment for total energy intake and homeostasis model assessment of insulin resistance (HOMA-IR). Our current study also indicated a positive correlation between frequency of fast eating and MASLD, after adjustment for all available confounding factors such as sex, age, demographics, smoking and drinking status, BMI and clinical metabolic parameters. We further performed a meta-analysis to clarify the relationship between MASLD and fast eating after adjustment for the effect of body weight and/or calorie intake. The result of meta-analysis was consistent with our finding that fast eating was independently associated with MASLD, with an estimated pooled OR (95%CI) of 1.22 (1.07–1.39). The pooled ORs remained statistically significant in the leave-one-out sensitivity analysis, indicating the reliability of the results. Thus, eating fast might regulate liver lipid metabolism directly.

Presumably, fast eating modulates the liver lipid metabolism by participating in the gut-liver-adipose axis. In other studies, eating speed proved to impact the postprandial response of the gut hormones, including orexigenic hormone ghrelin [37] and the anorexigenic peptides glucagon-like peptide-1 (GLP-1) and peptide YY (PYY) [27]. Many of gut peptides, especially ghrelin, showed interaction with the gastrointestinal microbiota [38, 39]. The metabolites of gut microbiota go directly into the liver through portal vein [40] and may contribute to progression of MASLD through the generation of free fatty acids [41]. Additionally, evidence suggests that GLP-1 in pancreatic β cells activate the AMPK signaling pathway, leading to the upregulation of liver X receptor (LXR)-mediated ATP-binding cassette transporter A1 (ABCA1) expression [42], which promotes the development of hepatosteatosis [43]. However, the evidence for either the two possible pathways is limited, and further investigation is required for the underlying mechanism of the direct impact of eating fast on MASLD development.

To the best of our knowledge, our current study is the largest nationwide multicenter study on the relationship between fast eating and the risk of MASLD. However, the information on energy intake and dietary composition was not available in our study. Although we included geographical background in the regression model, which was strongly related to dietary pattern [44], the confounding effect of calorie intake on the relationship between eating speed and risk of MASLD could not be completely ruled out. Additionally, all the reviewed studies were done in Asians and the definition of fast eating was not uniform, further studies were still required to expand the conclusion to participants from different ethnicities using the same criteria of eating speed categories. Last but not least, the cross-sectional analysis of the association between frequency of fast eating and MASLD did not permit an evaluation of the causal relationship, which still requires further prospective studies.

## Conclusions

In conclusion, the current study, along with the meta-analysis, indicated that frequently eating fast is positively associated with a higher risk of developing MASLD. Further research is required to uncover the underlying mechanisms behind the association, to explore the relationship between eating speed and liver fibrosis, and to investigate whether the improvement of eating habit, such as fast eating, is beneficial.

### Data sharing

Data described in the manuscript, code book, and analytic code will be made available from the corresponding author upon reasonable request.

### Supplementary information

Supplementary Materials
