# Supplementary material for: Association between fast eating speed and metabolic dysfunction-associated steatotic liver disease: a multicenter cross-sectional study and meta-analysis
Source: Nutr Diabetes. 2024 Aug 14;14:61. doi: 10.1038/s41387-024-00326-x (PMC11324733; doi:10.1038/s41387-024-00326-x)
Supplement: Supplementary file 1 — Supplementary Materials [file 41387_2024_326_MOESM1_ESM.pdf]

Association between fast eating speed and metabolic dysfunction-associated steatotic liver disease: A multicenter cross-sectional study and meta-analysis

SUPPLEMENTARY TABLES

Table S1. Participants characteristics by MASLD status

Characteristics[a]	Total(n=1965)	Controls (n=1006)	MASLD (n=959)	P-value[b]
Women, n(%)	988 (50.3)	543 (54.0)	445 (46.4)	0.001
Age,yr	52.78 ±13.65	52.34 ±14.46	53.25 ±12.72	0.143
Smoker, n(%)	542 (27.6)	294 (29.2)	248 (25.9)	0.106
Alcohol-drinker, n(%)	446 (22.7)	221 (22.0)	225 (23.5)	0.462
Glucose metabolism, n(%)				<0.001
NGT	494 (25.1)	354 (35.2)	140 (14.6)	
IGR	283 (14.4)	133 (13.2)	150 (15.6)	
T2DM	1188 (60.5)	519 (51.6)	669 (69.8)	
North, n(%)	896 (45.6)	396 (39.4)	500 (52.1)	<0.001
BMI, kg/m²	24.80 ±3.61	23.51 ±3.16	26.14 ±3.57	<0.001
SBP, mmHg	130.25 ±19.64	127.97 ±20.84	132.64 ±18.01	<0.001
DBP, mmHg	80.40 ±11.27	78.53 ±10.96	82.37 ±11.27	<0.001
WC, cm	87.93 ±10.76	91.19 ±10.08	84.83 ±10.46	0.001
HBA1C, %	6.90 (5.80- 9.00)	6.40 (5.60- 8.30)	7.50 (6.10- 9.35)	<0.001
FBG, mmol/L	6.11 (5.26- 7.90)	5.60 (5.10- 7.34)	6.60 (5.50- 8.60)	<0.001
2hPBG, mmol/L	11.42 (7.03- 16.60)	9.09 (6.39- 15.50)	13.27 (8.60- 17.50)	<0.001
TC, mmol/L	4.91 ±1.28	4.74 ±1.26	5.09 ±1.28	<0.001
TG, mmol/L	2.04 ±2.22	1.53 ±1.36	2.57 ±2.75	<0.001
HDL-c, mmol/L	1.24 ±0.40	1.30 ±0.42	1.17 ±0.36	<0.001
LDL-C, mmol/L	2.85 ±0.95	2.75 ±0.94	2.94 ±0.95	<0.001
ALT, U/L	18.00 (12.83- 28.00)	16.00 (11.00- 23.19)	21.00 (15.00- 32.00)	<0.001
AST, U/L	20.00 (16.00- 27.00)	19.00 (15.00- 25.00)	22.00 (17.00- 30.00)	<0.001
GGT, U/L	27.00 (16.00- 47.00)	21.00 (13.67- 38.25)	33.00 (20.96- 55.14)	<0.001
UA, µmol/L	296.84 ±97.80	283.74 ±94.62	310.52 ±99.24	<0.001

[a] Data are in n (%), means±SD or median (25th- 75th percentile), as appropriate.
[b] T test, Mann-Whitney test or chi-squared test

MAFLD, metabolic dysfunction-associated steatotic liver disease; NGT, normal glucose tolerance; IGR, impaired glucose regulation; T2DM, type 2 diabetes mellitus; BMI, body mass index; SBP, systolic blood pressure, DBP, diastolic blood pressure; WC, waist circumference; HbA1C, glycated hemoglobin; FBG, fasting blood glucose; 2hPBG, 2-hour postload blood glucose in oral glucose tolerance test; TC, total cholesterol; TG, triglyceride; HDL-c, high density lipoprotein cholesterol; LDL-c, low density lipoprotein cholesterol; ALT, alanine aminotransferase; AST, aspartate aminotransferase; GGT, gamma-glutamyl transferase; UA, uric acid

Table S2. Stratified ORs (95% CIs) for MASLD according to frequency categories of fast eating

	≤1 time/mo	≤1 time/wk	≥2 times/wk	P for trend	P for interaction
**Stratified by[a]**					
**Sex**					**0.257**
Men	Reference	1.15 (0.75-1.79)	1.42 (0.84-2.40)	0.179	
Women	Reference	0.95 (0.58-1.57)	2.25 (1.34-3.77)	0.007	
**Region**					**0.213**
South China	Reference	0.88 (0.55-1.42)	1.40 (0.81-2.41)	0.419	
North China	Reference	1.40 (0.87-2.24)	2.07 (1.24-3.44)	0.004	
**Smoking**					**0.935**
Non-smoker	Reference	1.18 (0.79-1.75)	1.80 (1.13-2.85)	0.014	
Smoker	Reference	0.95 (0.52-1.74)	1.82 (1.01-3.31)	0.082	
**Glucose status**					**0.194**
NGT	Reference	0.46 (0.14-1.56)	1.75 (0.49-6.31)	0.876	
IGR	Reference	1.75 (0.80-3.83)	3.82 (1.68-8.67)	0.001	
T2DM	Reference	0.92 (0.62-1.38)	1.42 (0.90-2.24)	0.241	
**BMI**					**0.248**
< 25 kg/m²	Reference	1.20 (0.78-1.84)	2.30 (1.41-3.76)	0.002	
≥25 kg/m²	Reference	0.90 (0.55-1.48)	1.51 (0.89-2.56)	0.219	

[a] Adjusted for age, sex, smoking status and drinking status, region, BMI, WC, HB1AC, FBG, TC, TG, HDL-c, LDL-c. ALT, AST, GGT, UA.

NGT, normal glucose tolerance; IGR, impaired glucose regulation; T2DM, type 2 diabetes mellitus; BMI, body mass index; WC, waist circumference; HbA1c, glycated hemoglobin; FBG, fasting blood glucose; 2hPBG, 2-hour postload blood glucose in oral glucose tolerance test; TC, total cholesterol; TG, triglyceride; HDL-c, high density lipoprotein cholesterol; LDL-c, low density lipoprotein cholesterol; ALT, alanine aminotransferase; AST, aspartate aminotransferase; GGT, gamma-glutamyl transferase; UA, uric acid.

Table S3. Summary of the characteristics and results from included articles

Study	Study Design	Methods	Sample Size & population	Age	Eating Speed Category	Reference	Unadjusted OR	Fairly Adjusted OR	Additional Information	Adjusted covariates
*Nishi, et al, 2016	Retrospective Cohort	Self-reported Eating Speed; NAFLD: FLI scores ≥60	n= 2,254 Japanese adults	48.0±10.0	Fast	Slow/moderate	1.58 (0.90-2.77)	1.33 (0.74-2.37)	NA	Sex, age, smoking, physical activities, metabolic status, prefecture.
*Lee, et al, 2016	Cross-sectional	Self-reported Eating Speed; NAFLD: Sonographic examination	n= 7,917 Korean adults	46.3 ± 12.9 (Normal group)<br>52.1 ± 11.9 (NAFLD group)	10≤ and <15 min;<br>5≤ and <10 min;<br><5 min	> 15 min	1.36 (1.17–1.59)<br>2.06 (1.77–2.39)<br>3.01 (2.44–3.71)	1.12 (0.87–1.43)<br>1.42 (1.11–1.81)<br>1.81 (1.24–2.63)	More pronounced effect found in the subgroup with BMI < 25 kg/m2	Sex, age, BMI, total energy intake, smoking, physical activity, biochemical measurements
*Cao, et al. 2020	Cross-sectional	Self-reported Eating Speed; NAFLD: Sonographic examination	n = 23,611 Chinese adults	38.6 ± 1.33 (Normal group)<br>42.3 ± 1.32 (NAFLD group)	Medium (16-20 min);<br>Relatively fast (11-15 min);<br>Very fast (≤10 min)	Slow (> 20 min)	1.52 (1.31-1.77)<br>2.09 (1.80-2.42)<br>2.65 (2.25-3.23)	1.06 (0.89-1.28)<br>1.08 (0.90-1.31)<br>1.04 (0.84-1.28)	NA	Sex, age, BMI, total energy intake, smoking, drinking, socioeconomic status, depressive symptoms,

										sleeping status, dietary patterns
*Iwai, et al. 2023	Cross-sectional	Self-reported Eating Speed; NAFLD: L/S attenuation	n = 450 Japanese adults	55 (45-62) (Normal group) 56 (45-69) (NAFLD group)	Fast	Not Fast	1.785 (1.036-3.075)	1.231 (0.649-2.336)	NA	Sex, age, BMI, chewing status, biochemical measurements
Takahashi, et al. 2020	Cross-sectional	Self-reported Eating Speed; NAFLD: Sonographic examination	n = 308 Japanese type 2 diabetes patients	66.8±10.6	Moderate; Fast	Slow	2.10 (0.97–4.54) 2.64 (1.25–5.56)	1.42 (0.59–3.44) 2.10 (0.90–4.91)	More pronounced effect found in male group	Sex, age, energy intake, smoking, exercise, biochemical measurements
Hsieh, et al. 2011	Cross-sectional	Self-reported Eating Speed; NAFLD: Sonographic examination	n = 8240 men<br>n = 2955 women<br>Japanese adults	51.6±9.4 (Men)<br>52.9±10.1 (Women)	Rapid	Not rapid	NA	Men: 1.69 (1.52-1.88)<br>Women: 1.62 (1.24-2.10)	NA	Age
Mansour-Ghanaei, et al. 2019	Cross-sectional	Self-reported Eating Speed; NAFLD: Sonographic examination	n = 630 Iranian adults	46.99 ± 7.33	Fast	Not Fast	1.40 (1.02-1.92)	NA	NA	NA

*included in the meta-analysis

Table S4. Quality Assessment of the Studies by Newcastle-Ottawa Scale (NOS)

		Retrospective Cohort	Cross-sectional					
		Nishi, 2016, Japan	Takahashi, 2020, Japan	Lee, 2016, Korea	Cao, 2020, China	Iwai, 2023, Japan	Hsieh, 2011, Japan	Mansour-Ghanaei, 2019, Iran
**s**	**1**	1	1	1	1	1	1	1
	**2**	1	1	1	1	1	1	1
	**3**	1	0	1	1	1	1	1
	**4**	0	1	1	1	1	1	1
**Comparability**	**1**	2	2	2	2	2	1	0
**Exposure**	**1**	0	1	1	1	1	1	1
	**2**	1	1	1	1	1	1	1
	**3**	0	0	0	0	0	0	0
**Total Points**		6	7	8	8	8	7	6
**Quality Evaluation**		Moderate	Moderate	High	High	High	Moderate	Moderate

Table S5. Quality Assessment of 6 cross-sectional studies the Agency for Healthcare Research and Quality (AHRQ) scale

	Takahashi, 2020, Japan	Lee, 2016, Korea	Cao, 2020, China	Iwai, 2023, Japan	Hsieh, 2011, Japan	Mansour-Ghanaei, 2019, Iran
**1**	1	1	1	1	1	1
**2**	1	1	1	1	1	1
**3**	1	1	1	1	1	1
**4**	1	1	1	1	1	1
**5**	1	1	1	1	1	1
**6**	0	1	1	1	1	0
**7**	1	1	1	1	1	1
**8**	1	1	1	1	1	0
**9**	0	1	1	0	0	0
**10**	0	0	0	0	0	0
**11**	0	0	0	0	0	0
**Total Points**	7	9	9	8	8	6
**Quality Evaluation**	Moderate	High	High	High	High	Moderate

SUPPLEMENTARY FIGURES

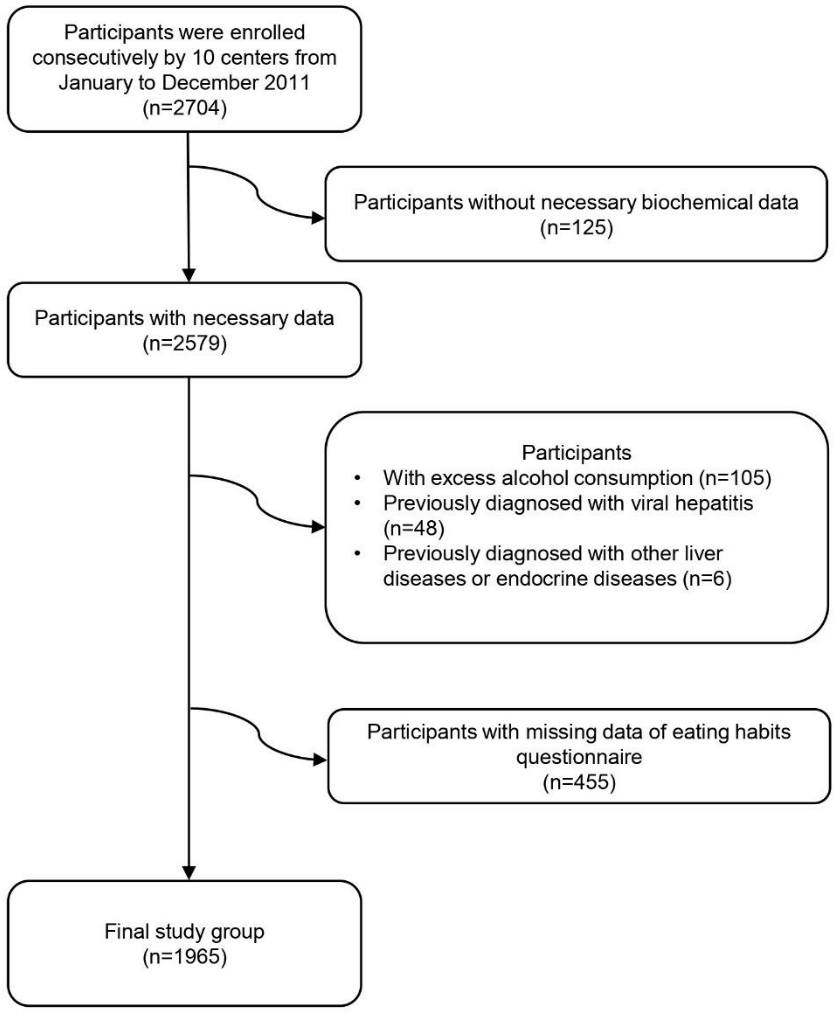

Fig. S1 - Flow diagram of study patients

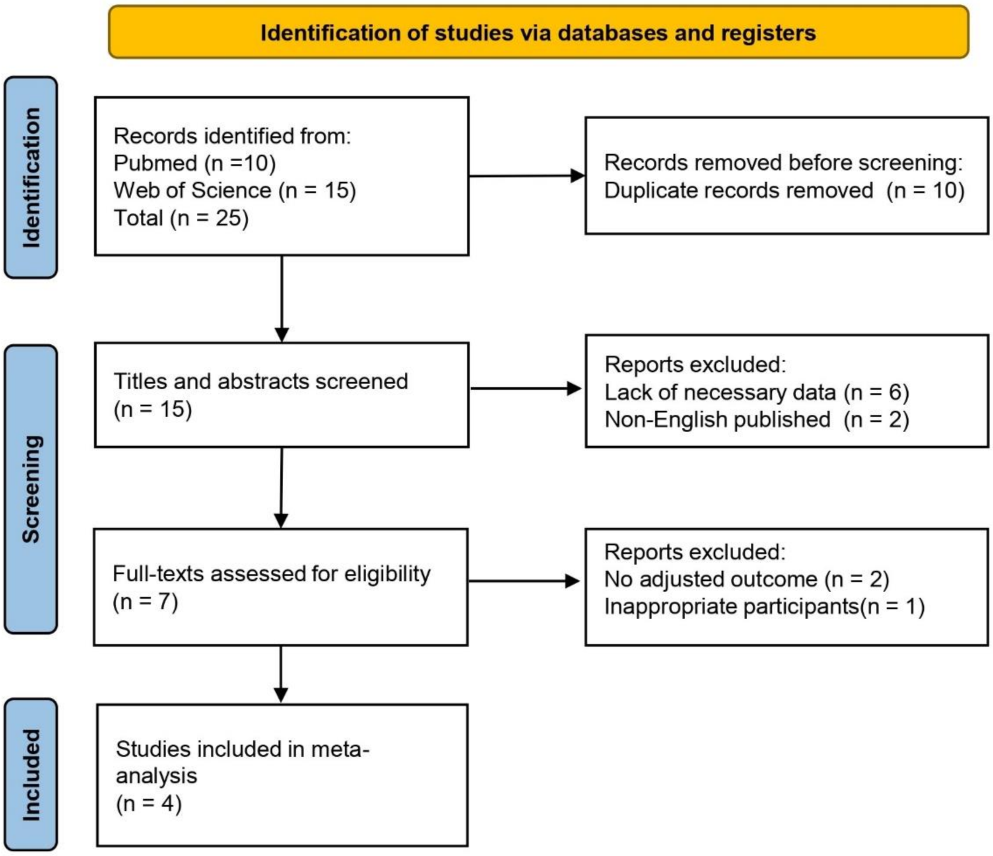

Fig. S2 - Flow diagram outlining the selection of studies included in the meta-analysis

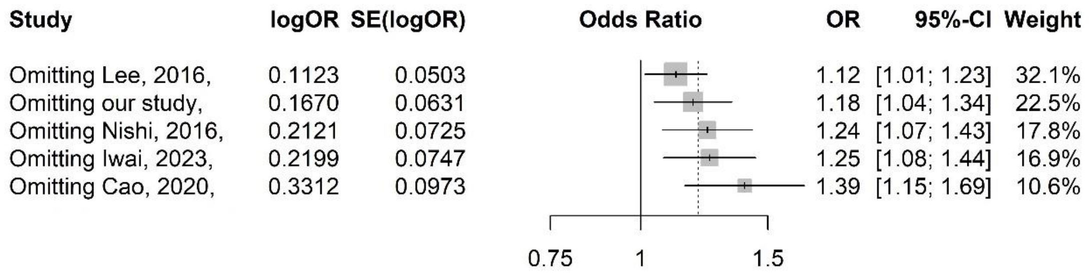

Fig. S3 - Forest plot for the leave-one-out sensitivity analysis of the meta-analysis.
